# A Reverse-Osmosis Model of Apoptotic Shrinkage

**DOI:** 10.3389/fcell.2020.588721

**Published:** 2020-10-23

**Authors:** Priyanka S. Rana, Michael A. Model

**Affiliations:** Department of Biological Sciences, Kent State University, Kent, OH, United States

**Keywords:** cytoskeleton, cytoskeletal contraction, apoptotic volume decrease, intracellular pressure, osmolytes, potassium channels

## Abstract

The standard theory of apoptotic volume decrease (AVD) posits activation of potassium and/or chloride channels, causing an efflux of ions and osmotic loss of water. However, in view of the multitude of possible channels that are known to support apoptosis, a model based on specific signaling to a channel presents certain problems. We propose another mechanism of apoptotic dehydration based on cytoskeletal compression. As is well known, cytoskeleton is not strong enough to expel a substantial amount of water against an osmotic gradient. It is possible, however, that an increase in intracellular pressure may cause an initial small efflux of water, and that will create a small concentration gradient of ions, favoring their exit. If the channels are open, some ions will exit the cell, relieving the osmotic gradient; in this way, the process will be able to continue. Calculations confirm the possibility of such a mechanism. An increase in membrane permeability for water or ions may also result in dehydration if accompanied even by a constant cytoskeletal pressure. We review the molecular processes that may lead to apoptotic dehydration in the context of this model.

## Introduction

The remarkable and often the most noticeable feature of apoptosis is a decrease in cell size. While many other characteristics of apoptosis are often cell- and stimulus-dependent, shrinkage occurs very reliably. Numerous publications investigating various aspects of apoptotic volume decrease (AVD) have appeared in the 1990–2000s and resulted in a significant progress in our understanding of this phenomenon.

When apoptosis was first recognized as a distinct type of cell death, it was dubbed “shrinkage necrosis” (Kerr, 1971) because cells undergoing this type of death become smaller. This observation was surprising because, when exposed to an unfavorable environment, cells are expected to accumulate water, increase their volume and eventually lose membrane integrity. Water accumulation is a natural response to cessation of the Na^+^-K^+^ pump activity (Armstrong, 2003) and is a hallmark of unregulated necrotic death (Zong and Thompson, 2006). If the opposite occurs during apoptosis, one is tempted to conclude that cell shrinkage is a purposeful response.

AVD occurs early in apoptosis, often before caspase activation (Maeno et al., 2000). It can continue for several hours (Maeno et al., 2000; l’Hoste et al., 2010; Poulsen et al., 2010; Kasim et al., 2013), and cells often reduce their volume by 10–20% (Model, 2014). Sometimes, application of potassium or chloride channel inhibitors simultaneously with an apoptotic stimulus prevents caspase activation as well as all other signs of apoptosis (Maeno et al., 2000; Wei et al., 2004). These facts have been interpreted in the sense that either the loss of water or the loss of potassium is a necessary step in apoptosis development.

**FIGURE 1:**
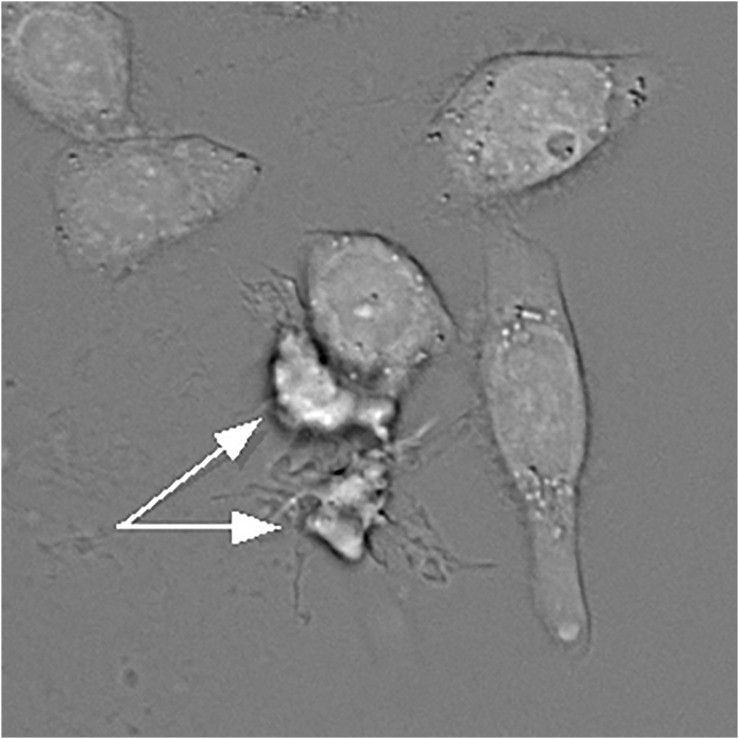
A bright-field transmission image of HeLa cells treated with actinomycin D. Two apoptotic and apparently dehydrated HeLa cells indicated by the arrows have much darker borders. However, this feature alone can only serve as a suggestion, but not as a definitive proof of dehydration. Water content can be accurately quantified under a transmission microscope as described by Mudrak et al. (2018).

It is worth noting that a decrease in apoptotic cell size can be brought about by two mechanisms, dehydration and fragmentation. Because of the sensitivity of shrinkage to potassium and chloride, dehydration has received much more attention in the literature and in effect has become synonymous with AVD. But these two paths are not always easy to distinguish from one another, and not every instance of cell volume decrease should automatically be attributed to dehydration (Model, 2014; Model et al., 2018). Dehydration can often be surmized (though not definitively established) by prominent cell borders in bright-field transmission images (Figure 1). It can been more rigorously demonstrated by buoyant density centrifugation (Wyllie and Morris, 1982; Yamada and Ohyama, 1988; Cohen et al., 1992; Patterson et al., 1995; Yurinskaya et al., 2005) or by combined phase/volume measurements (Model and Schonbrun, 2013; Model et al., 2018). A loss of osmotically active ions in UV-irradiated U937 cells with preservation of the amount of phosphorus (Fernández-Segura et al., 1999) can be taken as another evidence of dehydration. Apoptotically-induced activation of outward Cl^–^ currents (Porcelli et al., 2004; Okada et al., 2006; Ernest et al., 2008) also indirectly supports the notion of water loss since chloride is, theoretically, the main correlate of intracellular water (Armstrong, 2003; Fraser and Huang, 2007).

But chloride cannot leave the cell alone, without being accompanied by a cation (potassium), and the outward electrochemical drive for potassium is usually stronger than that for chloride. Thus, the standard model of AVD presumes an early activation of potassium channels, which stimulate an efflux of chloride; the resulting loss of osmolytes leads to osmotic dehydration (Yu, 2003; Lang et al., 2005; Bortner and Cidlowski, 2007; Lang and Hoffmann, 2012; Kondratskyi et al., 2015). One general difficulty with this theory has already been pointed out by other authors. “Very different types of K^+^ channels have been implicated in activation of apoptosis, like voltage-gated K_v_ channels, ATP-regulated K_ATP_ channels, two-pore K^+^ channels, several types of Ca^2+^-dependent K^+^ channels and others. The variety of K^+^ channels that have been implicated in apoptosis suggests that in principle any type of K^+^ channel can support apoptosis, but it is not entirely clear how this can operate as a specific switch driving cells into regulated cell death” (Kunzelmann, 2016). Indeed, one would expect that such a universal phenomenon as apoptotic dehydration should have a more universal reason than relying on activation of specific channels present in each particular cell type.

### Cytoskeleton-Driven, Ion-Dependent Dehydration

Here we wish to propose an alternative, or rather a complementary, mechanism of dehydration. It is not only ions that can drive water, but water can also drive ions through whatever channels happen to be available. One possible scenario would involve cytoskeleton-mediated intracellular pressure (IP).

IP in animal cells is conceptually similar to turgor in plant cells, although much smaller in magnitude—from tens to thousands Pa (Chengappa et al., 2018). The existence of intracellular pressure is evident from membrane blebs—the areas where membrane-cytoskeleton linkages mediated by numerous lipid-protein and protein-protein interactions (Doherty and McMahon, 2008) are weakened, and membrane forms protruding bubbles (Dai and Sheetz, 1999). Such blebs are frequently observed in apoptotic cells.

IP is believed to result from contraction of the actomyosin cytoskeleton. Since cell membranes are permeable to water, the existence of hydrostatic pressure implies that cells with stable water content must maintain a slight excess of intracellular osmolarity to keep water in balance. This is analogous to the balance of the Starling forces in blood capillaries.

We hypothesize that the balance of hydrostatic and osmotic forces becomes disrupted in apoptotic cells due to an increased cytoskeletal tension. Cytoskeleton has been long dismissed as a possible dehydration factor because the osmotic pressure that needs to be overcome to push water out of the cell is much stronger than any forces that can develop within the cytoskeleton; thus, researchers have come to believe that it is impossible for the cytoskeleton to produce any substantial water loss (Janmey, 1998). However, the situation would change radically if ion transport is taken into consideration. Suppose that a cell, initially at equilibrium, has developed a higher IP. This excess of pressure would result in a small water efflux, as in reverse osmosis, which, in turn, would create a slight increase in intracellular concentration of ions. If ion channels were blocked, water efflux would stop at this point because cytoskeleton would not be able to push against the ever-increasing concentration difference. But if ion channels were open, some ions would exit the cell down the concentration gradient (also aided by an excess of hydrostatic pressure inside). That would relieve the osmotic resistance and enable the cytoskeleton to push a little further, so that shrinkage would be able to continue.

As the following calculations show, the balance can be disrupted not only by an increase in hydrostatic pressure, but also by an increase in membrane permeability for ions (which makes the model equivalent to the standard one) or for water.

We will first describe the model in quantitative terms and then will discuss the experimental evidence for the cytoskeletal mechanism.

## Theory

Shrinkage rate due to cytoskeletal compression can be estimated as follows.

Consider a cell with volume V immersed in a solution with osmolarity C_0_; the internal concentration of osmolytes is C_i_ = C_0_ + ΔC, where ΔC is a small positive number. Additional cytoskeletal pressure S (which can be expressed in equivalent concentration units by dividing physical pressure by RT, which equals to 2.5⋅10^6^ Pa⋅L⋅mol^–1^ at 300K) causes an efflux of water and cell volume decrease, according to:

(1) $$\frac{1}{\rho }\,\frac{\text{dV}}{\text{dt}}=-\left[S-\left({\text{C}}_{\text{i}}-{\text{C}}_{\text{o}}\right)\right]\,{\text{AP}}_{\text{w}}=-\left(S-△\,\text{C}\right)\,{\text{AP}}_{\text{w}}$$

where ρ is the molar volume of water (18 cm^3^/mol), A is the surface area of the cell (cm^2^), and P_w_ is membrane permeability for water (cm/s). We can assume for simplicity that S, A, and P_w_ remain constant and only ΔC changes with time. Time-dependent changes in ΔC result from two factors: a decrease in the cell volume V (cm^3^) and an efflux of the osmolyte. If the total molar amount of the osmolyte present in the cell is N (mol), then:

(2) $$\frac{{\text{dC}}_{\text{i}}}{\text{dt}}=\frac{\text{d}}{\text{dt}}\,\left(\frac{\text{N}}{\text{V}}\right)=\frac{\text{d}}{\text{dt}}\,\frac{\mathrm{VdN}-\mathrm{NdV}}{{\text{V}}^{2}}=\frac{\text{dN}/\text{dt}}{\text{V}}-\text{N}\,\frac{\text{dV}/\text{dt}}{{\text{V}}^{2}}=\frac{\text{dN}/\text{dt}}{\text{V}}-{\text{C}}_{\text{i}}\,\frac{\text{dV}/\text{dt}}{\text{V}}$$

Time derivative of the volume dV/dt is expressed by Eq. 1, and the change in the amount of osmolyte dN/dt is expressed through membrane permeability for the osmolyte:

(3) $$\frac{\text{dN}}{\text{dt}}=-\left(S+△\,\text{C}\right)\,{\text{AP}}_{\text{i}}$$

Since

$$\frac{{\text{dC}}_{\text{i}}}{\text{dt}}=\frac{\text{d}\,△\,\text{C}}{\text{dt}}$$

we obtain:

(4) $$\frac{\text{d}\,△\,\text{C}}{\text{dt}}=-\frac{S+△\,\text{C}}{\text{V}}\,{\text{AP}}_{\text{i}}+\frac{{\text{C}}_{0}+△\,\text{C}}{\text{V}}\,\left(S-△\,\text{C}\right)\,\rho \,{\text{AP}}_{\text{w}}$$

We can further assume that the process has reached a quasi-steady state, when ΔC remains nearly constant; this allows us to estimate the rate of shrinkage by equating $\frac{\text{d}\,△\,\text{C}}{\text{dt}}$to zero. By neglecting quadratic terms and taking into account that C_0_ ≫ S, we find that, in a steady state:

(5) $$△\,\text{C}\approx \text{S}\,\frac{{\text{C}}_{0}\,\rho \,{\text{P}}_{\text{w}}-{\text{P}}_{\text{i}}}{{\text{C}}_{0}\,\rho \,{\text{P}}_{\text{w}}+{\text{P}}_{\text{i}}}$$

For an impermeant membrane (P_i_ = 0), the concentration difference becomes equal to S.

To find the rate of volume change, we substitute Eq. 5 into Eq. 1 and obtain:

(6) $$\frac{\text{dV}}{\text{dt}}=\frac{2\,\rho \,{\text{AP}}_{\text{i}}\,{\text{P}}_{\text{w}}}{{\text{C}}_{0}\,\rho \,{\text{P}}_{\text{w}}+{\text{P}}_{\text{i}}}\,\text{S}$$

As expected, shrinkage depends both on water and osmolyte permeabilities. For a spherical cell with radius r (cm), the relative rate of shrinkage will be:

(7) $$\frac{\text{dV}/{\text{V}}_{0}}{\text{dt}}=\frac{6\,\rho \,{\text{P}}_{\text{i}}\,{\text{P}}_{\text{w}}}{\text{r}\,\left({\text{C}}_{\text{o}}\,\rho \,{\text{P}}_{\text{w}}+{\text{P}}_{\text{i}}\right)}\,\text{S}$$

To evaluate this expression, we assume *r* = 5⋅10^–4^ cm and C_0_ = 10^–4^ mol/cm^3^ and use some literature values for the other parameters: P_i_ = 10^–5^ cm/s (Strickholm, 1981), P_W_ = 2⋅10^–3^ cm/s (Farinas et al., 1997), and S = 1,000 Pa (Chengappa et al., 2018), which correspond to 10^3^ Pa/(2.5⋅10^6^ Pa⋅L⋅mol^–1^) = 4⋅10^–7^ mol/cm^3^. That gives us the shrinkage rate of more than 40% per hour. Even though the parameters have been chosen rather arbitrarily, this estimate demonstrates that cytoskeleton-driven shrinkage is possible.

## Discussion

The standard theory of the AVD postulates that some yet unidentified early apoptotic reactions alter membrane permeability in such a way that intracellular osmolytes begin to come out of the cell. Because potassium is the only major ion with electrochemical gradient strongly favoring its exit, it is best suited for the role. However, leakage of potassium alone would immediately cause hyperpolarization that would terminate the process. To keep electroneutrality, potassium can be replaced with sodium, but that would fail to produce any osmotic imbalance. The only way inorganic ions can cause shrinkage is when the exit of potassium exceeds the entry of sodium, and the charge difference is balanced by the loss of chloride.

The results with ionophores agree with this reasoning. Specific potassium ionophore valinomycin causes cell volume loss only when anion exchange is allowed (Dise and Goodman, 1985), but since cells are normally permeable for chloride to some extent, application of valinomycin to intact cells results in a volume decrease (Rana et al., unpublished). A detailed analysis of ion and water balance in the presence of various membrane channels and transporters (Vereninov et al., 2004, 2007) confirms that an increase in potassium permeability may cause AVD, especially if accompanied by a reduced activity of the Na^+^-K^+^ pump. To our knowledge, IP has not been included in any of the previous models.

At the same time, the idea that hydrostatic pressure may affect cell volume is not entirely new. Bereiter-Hahn and Strohmeier (1987) suggested that calcium-dependent intracellular pressure is strong enough to prevent osmotic fluxes. However, that effect was only observed in hypotonic media, and therefore the intracellular pressure in their case was rather a passive resistance to stretch, which is different from active compression that we are hypothesizing. Another model of cell volume regulation assumes that changes in cell volume affect membrane tension and mechanosensitive channels, providing a negative feedback for volume maintenance (Jiang and Sun, 2013).

We approached the problem from a different angle. Equation (6) does not aim to describe a specific molecular process; we omit the membrane potential from the picture, and the nature of the membrane-permeant osmolyte is not even specified. Thus, our model is different from the detailed descriptions of osmotic balance found in the works of other authors (Armstrong, 2003; Fraser and Huang, 2004; Vereninov et al., 2004, 2007). Nevertheless, our result proves that cytoskeletal compression is in principle capable of slowly expelling water if accompanied by an efflux of osmolytes. Equation (6) predicts that the rate of shrinkage should be proportional to IP; it should also be an increasing function of ion and water permeabilities and a decreasing function of external osmolarity. All these trends can be subject to experimental test.

### Intracellular Pressure

The dependence of the AVD rate on IP is the critical feature of our model; moreover, we suggest that IP increases during apoptosis. The frequent occurrence of apoptotic blebs indicates that this indeed may be the case, with one qualification that blebs may result either from an increase in IP or from loosening of membrane attachment to the cytoskeleton. Most likely, both factors are in operation, but only the first one is relevant to our model.

The only known mechanism of IP generation is actomyosin contraction (Mills et al., 1998; Hagmann et al., 1999; Miñambres et al., 2006; Tinevez et al., 2009). The force is created by non-muscle myosin II, whose activity is regulated by phosphorylation by various kinases (Vicente-Manzanares et al., 2009), most importantly by myosin light chain kinase (MLCK) and by Rho-associated protein kinase (ROCK; Amano et al., 2010). MLCK is controlled by calcium and, indeed, persistent calcium elevation is a typical feature of apoptosis (Hajnóczky et al., 2003; Orrenius et al., 2003). Furthermore, MLCK activation during apoptosis has been demonstrated directly (Mills et al., 1998; Petrache et al., 2003; Nusbaum et al., 2004). On the other hand, buffering of intracellular calcium did not prevent a decrease in forward light scatter from apoptotic Jurkat cells (Scoltock et al., 2000), nor did it affect the loss of potassium and chloride in staurosporine-treated HeLa cells (Dezaki et al., 2012). The significance of these negative results is unclear because cell volume has not been measured in these works directly. Other authors did associate calcium with AVD, but only through its stimulatory effect on calcium-dependent chloride channels (Hoffmann et al., 2015).

The role of ROCK in apoptosis has also been a subject of investigation, mostly in connection to bleb formation. Under normal conditions, ROCK is activated by Rho GTPase but, during apoptosis, ROCK is cleaved by caspases 2 and 3 with release of a constitutively active fragment (Ndozangue-Touriguine et al., 2008; Street and Bryan, 2011). ROCK activation is responsible for apoptotic blebbing and detachment of apoptotic bodies (Street and Bryan, 2011).

**FIGURE 2:**
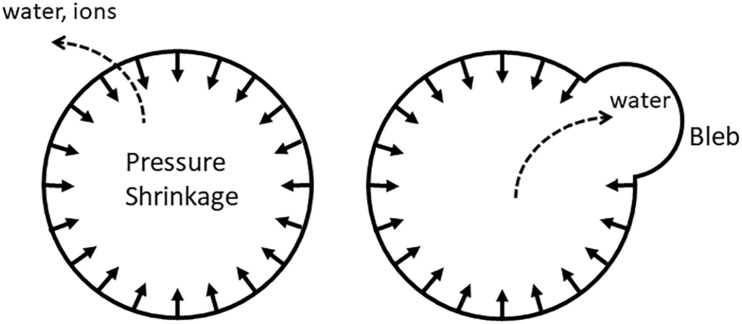
Uniform pulling on the membrane (either by the radial component of cortical tension or by non-cortical fibers oriented perpendicular to the membrane) produces IP that would slowly push water and ions out of the cell. When gaps in the cytoskeleton-membrane linkages are present, water is squeezed out into the bleb instead.

Conceivably, not every type of cytoskeletal tension would translate into an increased IP; the cytoskeleton must form a continuous shell and be firmly attached to the membrane, so that it would be effectively pulling the membrane inside; such actin-myosin rings are in fact observed during apoptosis (Coleman and Olson, 2002; Miñambres et al., 2006; Ndozangue-Touriguine et al., 2008; Povea-Cabello et al., 2017). The shape of the membrane may also play a role: the membrane must be sufficiently convex, so that cortical tension would have a radial component (Figure 2). Alternatively, inward pulling may be mediated by non-cortical filaments.

A more direct evidence in favor of the cytoskeletal hypothesis comes from two observations. Firstly, a myosin II inhibitor, blebbistatin, causes a slight but measurable increase in the volume of normal mitotic cells (Stewart et al., 2011). Our preliminary experiments on the effect of blebbistatin on apoptotic cells did show inhibition of water loss, but much more extensive studies would be needed for definitive conclusions. The problem with blebbistatin is that it inhibits apoptosis in general (Xiao et al., 2009; Street et al., 2010; Li et al., 2018; Chai et al., 2019). While such inhibition is compatible with the view that (1) cytoskeletal compression causes dehydration and that (2) dehydration is needed for apoptosis, one cannot claim that blebbistatin inhibits AVD if apoptosis is no longer present.

The second observation is that apoptotic water loss requires intact actin cytoskeleton. We subjected Madin-Darby Canine Kidney cells to a treatment with 2 mM staurosporine and measured intracellular water content, as described in Mudrak et al. (2018). After 3 h, cells lost 4.2% of their water (SEM 0.15 based on 45 cells analyzed in 3 separate experiments). However, the presence of 0.4 mM of the actin-depolymerizing drug cytochalasin D completely abrogated water loss (0.00 + 0.03, 31 cells in 3 experiments; *P* < 0.0001). A related finding has been reported by Bortner et al. (2008), who noticed that cytochalasin prevents potassium loss during apoptosis.

The other fact that agrees with the cytoskeletal model is that shrinkage does not occur in actively blebbing cells (Kasim et al., 2013; Gibbons et al., 2016), presumably because blebs provide an internal reservoir for water (Tinevez et al., 2009; Gibbons et al., 2016; Figure 2). Incidentally, blebbing in healthy cells also occurs with volume preservation (Langridge and Kay, 2006; Young and Mitran, 2010). On the other hand, application of blebbistatin or inhibition of ROCK by Y-27632 not prevent the development of cells with shrunken appearance (Kasim et al., 2013). However, neither water content nor IP have been determined in that study.

Unlike intracellular water, which can be quantified non-invasively under a regular transmission microscope (Mudrak et al., 2018), measuring IP in animal cells is a more challenging undertaking. The servo-null method that uses a microelectrode inserted into the cytoplasm (Kelly and Macklem, 1991; Petrie and Koo, 2014) may be the most direct and the least affected by the stiffness of the cytoskeleton because the needle penetrates past the cortical layer. A pressure-sensitive microinterferometer internalized by cells (Gómez-Martínez et al., 2013) is another interesting and apparently gentle technique for IP measurements.

### Ion Permeability

Permeability for ions should be an integral part of any dehydration scheme and does not represent a unique feature of our hypothesis. The value of 1.3^∗^10^–5^ cm/s used in our calculations has been reported for K^+^ in the resting axon (Strickholm, 1981). Even though some other published numbers are smaller (Costa et al., 1989; Fraser and Huang, 2004), an increase in potassium permeability and the loss of potassium from apoptotic cells have been convincingly demonstrated (Yu et al., 1997; Dallaporta et al., 1998), and this observation is the main buttress for the ionic theory of the AVD. Clearly, if ion permeability during apoptosis is sufficient to allow potassium out by electrochemical gradient alone, it should be all the more sufficient when electrochemical gradient is assisted by pressure. Volume-regulated anion channels (VRAC) that pass chloride and organic osmolytes also become activated during apoptosis (Osei-Owusu et al., 2018). Multiple ion fluxes are always interconnected (Vereninov et al., 2007), and the single P_i_ and C_0_ in Eq. 6 should be interpreted as cumulative parameters.

### Water Permeability

If dehydration is the essence of the AVD, it is only natural that water permeability should be a factor. The assumed value for water permeability, *P*_W_ = 2⋅10^–3^ cm/s, seems to be typical, although the numbers vary from 3 to 6 × 10^–4^ cm/s in MDCK cells (Farinas and Verkman, 1996; Zelenina and Brismar, 2000) to 0.1 cm/s in the rat proximal tubule (Preisig and Berry, 1985). When water permeability is high, Eq. 7 turns into:

(8) $$\frac{\text{dV}/{\text{V}}_{0}}{\text{dt}}=\frac{6\,\rho \,{\text{P}}_{\text{i}}\,{\text{P}}_{\text{w}}}{\text{r}\,\left({\text{C}}_{\text{o}}\,\rho \,{\text{P}}_{\text{w}}+{\text{P}}_{\text{i}}\right)}\,\text{S}\approx \frac{6\,{\text{P}}_{\text{i}}}{{\text{rC}}_{\text{o}}}\,\text{S}$$

and ion permeability becomes the only limiting factor.

The dependence of apoptosis on water permeability has been demonstrated by several authors using either chemical inhibition of aquaporins or by comparing similar cell types with different aquaporin expression (Jablonski et al., 2004; Flamenco et al., 2009; Younts and Hughes, 2009; Lakner et al., 2011; Zhang et al., 2011). To explain the relationship between apoptosis and aquaporins, Jablonski et al. (2004) suggested that, when aquaporins are blocked, the loss of potassium exceeds the loss of water, and a drop in potassium concentration causes apoptosis. Whether this is so or not, our main focus here is on the loss of water rather than on caspase activation and other degradative processes. The published results do not provide enough information to test Eq. 6, and more pointed and quantitative experiments would be desirable.

### Osmolarity

Cells adapt to a large range of osmolarities, and even the osmolarity of commercial DMEM media from different sources may vary from 230 to 360 mOsm. Although it is technically straightforward to manipulate osmolarity to test its effect on the AVD rate, varying levels of osmolarity may affect too many processes (Burg et al., 2007), and it would be difficult to guarantee that any changes in shrinkage rate are due to the described mechanism.

## Conclusion

The main claim of our hypothesis is that cytoskeletal pressure plays an active role in apoptotic water loss. The attractiveness of this proposition is that cytoskeletal rearrangements appear to be a general feature of apoptosis and therefore, this mechanism would not have to rely on specific properties of ion channels. The effect of blebbistatin on cell volume (Stewart et al., 2011) and the dependence of AVD on actin show that the model is not in conflict with observations. Its more direct testing may proceed in two main directions.

(1) Demonstration of an increased IP during AVD. It is unclear whether the existing technology would be adequate for the task. For example, puncturing apoptotic cells with a needle may be difficult to perform, and the use of intracellular microinterferometer may not have been sufficiently developed.
(2) Manipulation of myosin activity by drugs. The previously mentioned difficulty with blebbistatin (namely, its interference with apoptosis) could possibly be circumvented by applying it at later stages, when apoptosis has become irreversible.

It is less clear if quantification of the dependence of the AVD rate on ion and water permeability to test Eq. 6 can be accurate enough to distinguish between this model and the model based on ion channel activation (Vereninov et al., 2004, 2007). Moreover, these mechanisms are not mutually exclusive, and both are likely to operate in parallel.

## Data Availability Statement

The raw data supporting the conclusions of this article will be made available by the authors, without undue reservation, to any qualified researcher.

## Author Contributions

PR performed the experiments and participated in writing the manuscript. MM conceived the model and wrote the manuscript. All authors contributed to the article and approved the submitted version.

## Conflict of Interest

The authors declare that the research was conducted in the absence of any commercial or financial relationships that could be construed as a potential conflict of interest.
